# Canadian dentists’ awareness and views on early childhood caries and its prevention and management

**DOI:** 10.3389/froh.2023.1268350

**Published:** 2024-01-08

**Authors:** Joshua Levesque, Suhird Ghotra, Betty-Anne Mittermuller, Daniella DeMaré, Victor H. K. Lee, Vivianne Cruz de Jesus, Olubukola O. Olatosi, Hamideh Alai-Towfigh, Robert J. Schroth

**Affiliations:** ^1^ Department of Preventive Dental Science, Dr. Gerald Niznick College of Dentistry, Rady Faculty of Health Sciences, University of Manitoba, Winnipeg, MB, Canada; ^2^ Children’s Hospital Research Institute of Manitoba, Winnipeg, MB, Canada; ^3^ Department of Oral Biology, Dr. Gerald Niznick College of Dentistry, Rady Faculty of Health Sciences, University of Manitoba, Winnipeg, MB, Canada; ^4^ Department of Pediatrics and Child Health, Max Rady College of Medicine, Rady Faculty of Health Sciences, University of Manitoba, Winnipeg, MB, Canada; ^5^ Shared Health Inc., Winnipeg, MB, Canada

**Keywords:** access to care, early childhood caries, early childhood oral health care, child, preschool, pediatric dentistry, survey

## Abstract

**Introduction:**

The Canadian Dental Association (CDA) recommends children visit a dentist within 6 months of the eruption of their first tooth or by 12 months of age. The aim of this study was to investigate Canadian dentists’ awareness and views on early childhood caries (ECC) and its prevention and management.

**Methods:**

This study analyzed a subset of questions relating to dentists’ knowledge of ECC and prevention strategies, from a national survey of general and pediatric dentists, commissioned by the CDA in 2013. Analyses included descriptive, bivariate, and multivariate analyses. A *p*-value of ≤0.05 was considered significant.

**Results:**

Three thousand two hundred thirty-two out of 14,747 dentists responded (response rate of 21.9%), with 95.1% having heard of ECC. Overall, 60.9% of respondents reported that they were comfortable providing treatment to children with ECC. Significant differences were found between the number of years in practice and whether dentists were or were not comfortable providing prevention (19.5 ± 12.6 years vs. 25.4 ± 12.1 years; *p* < 0.001) or treatment for patients with ECC (19.1 ± 12.7 years vs. 22.5 ± 12.3 years; *p* < 0.001). Pediatric dentists (OR = 6.92; 95% CI: 2.57, 18.61), female dentists (OR = 1.13; 95% CI: 1.03, 1.24), dentists practicing in smaller urban areas (OR = 1.17; 95% CI: 1.07, 1.28), and dentists who were aware of the CDA's position on ECC (OR = 1.26; 95% CI: 1.13, 1.41) were more likely to be comfortable providing treatment for children with ECC.

**Conclusions:**

While the majority of Canadian dentists have sufficient knowledge of ECC, not all are comfortable providing oral health care services to children at a young age. It is encouraging however, that most dentists are wanting additional oral health resources designed for education on ECC prevention for parents of young children.

## Introduction

In children <6 years of age, any past or current decay involving primary teeth is indicative of the chronic disease known as early childhood caries (ECC) (1). Unfortunately, ECC is common among Canadian children, even though it is theoretically preventable (2, 3). Consequences of untreated ECC can often include pain, malocclusion, and abscesses. Severe early childhood caries (S-ECC) has also been found to negatively impact oral health-related quality of life, nutrition, and growth (4–6). ECC is a complex multifactorial disease, however it can often be avoided with consistent practice of preventive oral health-related behaviours, such as maintaining infrequent sugar consumption and implementing an effective oral hygiene routine (7). Prenatal and early childhood dental visits serve as an avenue for dentists and dental hygienists to promote oral health and improve parents’ oral health knowledge (8, 9).

The Canadian Dental Association (CDA) recommends that children see a dentist within 6 months of the eruption of the first tooth or by one year of age, since dentists are likely able to identify patients at high-risk for future caries development at this stage (10). Additionally, early visits to the dental office can increase a child's comfort level in the dental chair and aid in the establishment of a dental home (11). Belonging to a dental home during early childhood allows for preventive care to be disseminated in the form of caries-risk assessment, topical fluoride applications, anticipatory guidance, and parental oral health education (12). Having these preventive measures in place during early childhood sets a strong foundation for good oral health throughout childhood and into adolescence. Studies suggest that the earlier a child receives preventive dental care, the less dental treatment they will require throughout childhood (13–15). However, a potential barrier in access to care for young children is dentists’ views on when a child's first dental visit should occur (3) and the willingness of dentists to provide services for infants and toddlers (16). It has been suggested that not all dentists accept patients at a young age, with parents and caregivers being told to come back when their child is older (17). A recent study has found that the majority of Canadian dentists do not recommend a child's first dental visit before one year of age, even though it is the official recommendation of the CDA (18).

Therefore, the purpose of this study was to report on the findings of a national survey of Canadian dentists about their knowledge and views on ECC and their preventive strategies used with expecting patients and parents of infants and young children.

## Methods

In January of 2013, the CDA commissioned Navigator Ltd. to administer a national survey of general and pediatric dentists to gain insight of their knowledge and attitudes towards a child's first dental visit, early preventive dental visits, and ECC. The survey was sent to a total of 14,747 registered dentists in Canada. General and pediatric dentists were sent email invitations to the electronic survey. Two follow-up emails were sent to increase participation. The survey collected demographic information and covered first dental visits, preventive strategies, and ECC. Using data from this survey, Alai-Towfigh et al. (18), from our research group, investigated dentists’ views on a child's first dental visit and provides more details on the methods of this survey.

This present investigation consisted of a secondary analysis of a subset of questions from the survey, with approval by the CDA. The University of Manitoba's Health Research Ethics Board provided ethics approval for this investigation. Only survey questions relating to dentists’ knowledge of ECC and views on prevention strategies for young patients and their caregivers were selected for this analysis. Other variables of interest included gender, number of years in practice, type of dentist, type of practice, and location of practice. Practices were categorized as being either solo private, group private, or non-private (including Non-Insured Health Benefits (NIHB) contract dentists, hospital-based clinic specialists and general practitioners (GPs), and university-based specialists and GPs) practices. Provinces were grouped into western provinces and territories (BC, AB, SK, MB, YT, NT, NU), central provinces (ON, QC), and eastern provinces (NS, NB, NL, PE). Location of practice was coded as either being in a census metropolitan area (CMA) or non-census metropolitan area (non-CMA) (CMAs are defined as areas having a population of >100,000 with at least 50,000 people living in the urban core, while non-CMAs are smaller urban areas having a population of <100,000). The analyses were conducted using Number Cruncher Statistical Software 2021 (Kaysville, Utah). Descriptive statistics were calculated, and data was analyzed, comparing dentists’ characteristics and their views on ECC. Analyses included chi-square tests and independent sample *t*-tests. For these analyses of dentists’ comfort in providing prevention and treatment for children with ECC, survey selections of “strongly agree” and “agree” were both considered as comfortable providing the service, while “strongly disagree”, “disagree”, and “neither agree nor disagree” were all considered as not comfortable providing the service. Multiple logistic regressions were conducted. Outcome variables of interest were dentists’ relative comfort in providing prevention and treatment for children with ECC. For all analyses, a *p*-value of ≤0.05 was considered statistically significant.

## Results

A total of 3,232 dentists participated, yielding a response rate of 21.9%. Overall, the majority of respondents were general dentists (96.6%) and had heard of ECC (95.1%). Of those who had heard of ECC, half reported themselves as being very familiar with the definition (50.9%). However, fewer respondents were very familiar with the definition of severe ECC (S-ECC) (34.3%). Despite dentists’ awareness of ECC, only 58.0% reported being aware of the CDA's position on ECC. Participants were asked to report on their comfort in providing oral health care for children with ECC. Most respondents said they were comfortable providing prevention (85.2%), while fewer respondents said they were comfortable providing treatment to children with ECC (60.9%) (Table 1).

**Table 1:** Practitioners’ knowledge and awareness of ECC.

Knowledge and awareness of ECC	No. of respondents (%)
Heard of ECC (*n* = 2,710)	
Yes	2,578 (95.1)
No	132 (4.9)
Familiar with the definition of ECC (*n* = 2,544)	
Very familiar	1,296 (50.9)
Somewhat familiar	1,090 (42.9)
Somewhat unfamiliar	126 (5.0)
Not at all familiar	32 (1.3)
Familiar with the definition of S-ECC (*n* = 2,544)	
Very familiar	873 (34.3)
Somewhat familiar	1,246 (49.0)
Somewhat unfamiliar	286 (11.2)
Not at all familiar	139 (5.5)
Aware of the CDA's position on ECC (*n* = 2,522)	
Yes	1,464 (58.0)
No	1,058 (42.0)
Aware of Provincial Dental Association's position on ECC (*n* = 2,495)	
Yes	1,342 (53.8)
No	1,153 (46.2)
Comfortable providing prevention for children with ECC (*n* = 2,584)	
Agree	1,512 (58.5)
Somewhat agree	690 (26.7)
Neither agree nor disagree	240 (9.3)
Somewhat disagree	100 (3.9)
Disagree	42 (1.6)
Comfortable providing treatment for children with ECC (*n* = 2,584)	
Agree	870 (33.7)
Somewhat agree	704 (27.2)
Neither agree nor disagree	358 (13.9)
Somewhat disagree	381 (14.7)
Disagree	271 (10.5)

Table 2 shows strategies used by participants for prevention and management of ECC. Of those who had heard of ECC, most respondents said they use topical fluorides in general to manage caries in their patients (70.4%), while nearly two-thirds (64.8%) of respondents said they specifically use fluoride varnish. More than half of the respondents who had heard of ECC said they place sealants on primary teeth of high-risk children under six years of age to prevent the disease (54.4%). Nearly all respondents who had heard of ECC said they talk to parents about how to prevent it (96.2%). Many, but not all respondents reported that they discuss key messages with patients that are expecting a child. Only 27.2% of respondents said they always discuss proper infant nutrition with patients that are expecting a child, while almost half said they sometimes do so (47.5%). Slightly fewer dentists said they always (18.9%) or sometimes (43.8%) provide patients with information on the role maternal/prenatal nutrition plays during pregnancy in an infant's oral health. A similar proportion of dentists said they always (24.3%) or sometimes (42.3%) provide preventive oral health information to expectant mothers about the transmissibility of cariogenic bacteria.

**Table 2:** Respondent's use of various strategies to prevent or manage ECC.

Prevention or management practice used	No. of respondents (%)
Use topical fluoride to manage ECC (*n* = 2,544)	
Yes	1,791 (70.4)
No	753 (29.6)
Use fluoride varnish to manage ECC (*n* = 2,544)	
Yes	1,649 (64.8)
No	895 (35.2)
Place sealants on primary teeth of high-risk children <6 years of age in order to prevent ECC (*n* = 2,544)	
Yes	1,383 (54.4)
No	1,161 (45.6)
Provide oral hygiene instruction, tooth brushing techniques for young children and topical fluoride or varnish treatment as necessary (*n* = 2,542)	
Often	2,138 (84.1)
Somewhat often	345 (13.6)
Rarely	37 (1.4)
Never	22 (0.9)
Use pit and fissure sealants on primary molars to help prevent cavities (*n* = 2,542)	
Often	532 (20.9)
Somewhat often	619 (24.4)
Rarely	1,002 (39.4)
Never	389 (15.3)
Talk to parents about how to prevent ECC (*n* = 2,544)	
Yes	2,446 (96.1)
No	61 (2.4)
Unsure	37 (1.5)
Discuss proper infant nutrition with my patients expecting a child (*n *= 2,522)	
Always	687 (27.2)
Sometimes	1,199 (47.5)
Never	636 (25.2)
Provide patients with information on the role maternal/prenatal nutrition plays during pregnancy in an infant's oral health (*n* = 2,522)	
Always	476 (18.9)
Sometimes	1,105 (43.8)
Never	941 (37.3)
Provide preventive oral health information to expectant mothers about the transmissibility of cariogenic bacteria (*n* = 2,522)	
Always	612 (24.3)
Sometimes	1,067 (42.3)
Never	843 (33.4)

Participants were asked if they were receiving enough current information on the prevention and management of ECC and if they would like additional oral health promotional resources designed to inform families on infant and toddler oral health (Table 3). More than a third of the respondents said they were not receiving enough current information on ECC (36.8%), and most said they would like additional oral health promotional resources for families (83.2%). Nearly all participants said they would discuss prevention of ECC if provided with appropriate educational materials (96.5%).

**Table 3:** Respondents’ needs for additional oral health resources.

Need for education resources	No. of respondents (%)
Receiving enough current information on the prevention and management of ECC. (*n* = 2,584)	
Agree	370 (14.3)
Somewhat agree	612 (23.7)
Neither agree nor disagree	652 (25.2)
Somewhat disagree	694 (26.9)
Disagree	256 (9.9)
Would like (additional) oral health promotional resources designed to inform families on infant and toddler oral health and how to prevent ECC. (*n* = 2,584)	
Agree	1,403 (54.3)
Somewhat agree	747 (28.9)
Neither agree nor disagree	287 (11.1)
Somewhat disagree	63 (2.4)
Disagree	84 (3.3)
Would discuss prevention of ECC if provided with appropriate educational materials. (*n* = 2,544)	
Yes	2,455 (96.5)
No	89 (3.5)

Table 4 shows the associations between respondent characteristics and whether they reported being comfortable providing prevention for patients with ECC or not. Analysis with chi-square tests showed that general dentists were significantly less likely to be comfortable providing prevention for patients with ECC than pediatric dentists (84.8% vs. 97.8%; *p* < 0.001). Male dentists were also found to be less likely to be comfortable providing prevention for patients with ECC than female dentists (80.5% vs. 91.8%; *p* < 0.001). Associations were found between the proportions of dentists working mainly in solo private, group private, and non-private practices that were comfortable providing prevention for patients with ECC (83.2% vs. 86.3% vs. 90.0%; *p* < 0.05). An association was also found between the proportions of dentists located in western provinces and territories (BC, AB, SK, MB, YT, NT, NU), central provinces (ON, QC), and eastern provinces (NS, NB, NL, PE) that were comfortable providing prevention for patients with ECC (86.0% vs. 83.7% vs. 89.2%; *p* < 0.05).

**Table 4:** Associations between respondent characteristics and awareness of ECC and their comfort in providing prevention for patients with ECC.

Variable	Agree	Disagree	*p*-value
Type of dentist			
General dentist (*n* = 2,495)	2,115 (84.8)	380 (15.2)	
Pediatric dentist (*n* = 89)	87 (97.8)	2 (2.2)	<0.001
Gender			
Male (*n* = 1,505)	1,212 (80.5)	293 (19.5)	
Female (*n* = 1,079)	990 (91.8)	89 (8.2)	<0.001
Type of practice			
Solo private practice (*n* = 1,094)	910 (83.2)	184 (16.8)	
Group private practice (*n* = 1,340)	1,157 (86.3)	183 (13.7)	
Non-private practice (*n* = 150)	135 (90.0)	15 (10.0)	0.022
Location of practice			
Census metropolitan area (*n* = 1,292)	1,094 (84.7)	198 (15.3)	
Non-census metropolitan area (*n* = 1,281)	1,098 (85.7)	183 (14.3)	0.46
Province/territory			
Western provinces and territories (BC, AB, SK, MB, YT, NT, NU) (*n* = 999)	859 (86.0)	140 (14.0)	
Central provinces (ON, QC) (*n* = 1,290)	1,080 (83.7)	210 (16.23)	
Eastern provinces (NS, NB, NL, PE) (*n* = 295)	263 (89.2)	32 (10.8)	0.041
Heard of ECC			
Yes (*n* = 2,458)	2,142 (87.1)	316 (12.9)	
No (*n* = 125)	59 (47.2)	66 (52.8)	<0.001
Familiar with the definition of ECC			
Yes (*n* = 2,307)	2,049 (88.8)	258 (11.2)	
No (*n* = 151)	93 (61.6)	58 (38.4)	<0.001
Familiar with the definition of S-ECC			
Yes (*n* = 2,042)	1,834 (89.8)	208 (10.2)	
No (*n* = 416)	308 (74.0)	108 (26.0)	<0.001
Aware of the CDA's position on ECC			
Yes (*n* = 1,464)	1,329 (90.8)	135 (9.2)	
No (*n* = 1,057)	825 (78.1)	232 (21.9)	<0.001
Aware of their Provincial Dental Association's position on ECC			
Yes (*n* = 1,409)	1,271 (90.2)	138 (9.8)	
No (*n* = 1,086)	859 (79.1)	227 (20.9)	<0.001

Table 5 shows the associations between respondent characteristics and whether they reported being comfortable providing treatment for patients with ECC or not. Similarly, general dentists were less likely than pediatric dentists (59.6% vs. 97.8%; *p* < 0.001) and male dentists were less likely than female dentists (56.8% vs. 66.6%; *p* < 0.001) to be comfortable providing treatment for patients with ECC. Associations were found between the proportions of dentists working mainly in solo private, group private, and non-private practices that were comfortable providing treatment for patients with ECC (57.5% vs. 62.6% vs. 68.7%; *p* < 0.01). Dentists practicing in CMAs were also less likely than dentists practicing in non-CMAs to be comfortable providing treatment (58.5% vs. 63.3%; *p* < 0.05).

**Table 5:** Associations between respondent characteristics and awareness of ECC and their comfort in providing treatment for patients with ECC.

Variable	Agree	Disagree	*p*-value
Type of dentist			
General dentist (*n* = 2,495)	1,487 (59.6)	1,008 (40.4)	
Pediatric dentist (*n* = 89)	87 (97.8)	2 (2.2)	<0.001
Gender			
Male (*n* = 1,505)	855 (56.8)	650 (43.2)	
Female (*n* = 1,079)	719 (66.6)	360 (33.4)	<0.001
Type of practice			
Solo private practice (*n* = 1,094)	632 (57.8)	462 (42.2)	
Group private practice (*n* = 1,340)	839 (62.6)	501 (37.4)	
Non-private practice (*n* = 150)	103 (68.7)	47 (31.3)	0.007
Location of practice			
Census metropolitan area (*n* = 1,292)	756 (58.5)	536 (41.5)	
Non-census metropolitan area (*n* = 1,281)	812 (63.4)	469 (36.6)	0.011
Province/territory			
Western provinces and territories (BC, AB, SK, MB, YT, NT, NU) (*n* = 999)	603 (60.4)	396 (39.6)	
Central provinces (ON, QC) (*n* = 1,290)	778 (60.3)	512 (39.7)	
Eastern provinces (NS, NB, NL, PE) (*n* = 295)	193 (65.4)	102 (34.6)	0.24
Heard of ECC			
Yes (*n* = 2,458)	1,534 (62.4)	924 (37.6)	
No (*n* = 125)	40 (32.0)	85 (68.0)	<0.001
Familiar with the definition of ECC			
Yes (*n* = 2,307)	1,481 (64.2)	826 (35.8)	
No (*n* = 151)	53 (35.1)	98 (64.9)	<0.001
Familiar with the definition of S-ECC			
Yes (*n* = 2,042)	1,342 (65.7)	700 (34.3)	
No (*n* = 416)	192 (46.2)	224 (53.8)	<0.001
Aware of the CDA's position on ECC			
Yes (*n* = 1,464)	1,003 (68.5)	461 (31.5)	
No (*n* = 1,057)	533 (50.4)	524 (49.6)	<0.001
Aware of their Provincial Dental Association's policy on ECC			
Yes (*n* = 1,409)	947 (67.2)	462 (32.8)	
No (*n* = 1,086)	577 (53.1)	509 (46.9)	<0.001

There was a significant difference between the mean number of years in practice for dentists that were comfortable providing prevention for patients with ECC and for dentists that were not (19.5 ± 12.6 years vs. 25.4 ± 12.1 years; *p* < 0.001). Similarly, a significant difference was found between the mean number of years in practice for dentists that were comfortable providing treatment for patients with ECC and for dentists who were not (19.1 ± 12.7 years vs. 22.5 ± 12.3 years; *p* < 0.001).

Multiple logistic regressions were performed. Table 6 shows the first regression, for which the outcome variable was the dentists’ relative comfort in providing prevention for children with ECC. Results showed that the odds of the dentists being comfortable with providing prevention for children with ECC decreased by 3% with each additional year in practice (OR = 0.97, 95% CI: 0.96, 0.98; *p* < 0.0001). Pediatric dentists were 2.12 times more likely to report comfort in providing prevention than general dentists (OR = 2.12, 95% CI: 1.04, 4.33; *p* = 0.039). Female dentists were 48% more likely to report comfort in providing prevention than male dentists (OR = 1.48, 95% CI, 1.28, 1,71; *p* < 0.0001). Dentists who reported having heard of ECC were 2.08 times more likely to report comfort in providing prevention (OR = 2.08, 95% CI: 1.69, 2.56; *p* < 0.0001). Dentists who were aware of the CDA's position on ECC were 28% more likely to report comfort in providing prevention (OR = 1.28, 95% CI: 1.10, 1.50; *p* = 0.002) and those who were aware of their own Provincial Dental Association's (PDA) position on ECC were 29% more likely to report comfort in providing prevention (OR = 1.29, 95% CI: 1.10, 1.50; *p* = 0.001).

**Table 6:** Multivariate logistic regression model with participant characteristics and awareness of ECC for their comfort in providing prevention for children with ECC.

Variable	Regression coefficient	Odds ratio	95% CI for odds ratios	*p*-value
Intercept	1.02	–	–	–
Years in practice	−0.03	0.97	0.96, 0.98	<0.0001
Pediatric dentist^a^	0.75	2.12	1.04, 4.33	0.039
Female^b^	0.39	1.48	1.28, 1.71	<0.0001
Group private practice^c^	−0.13	0.88	0.69, 1.12	0.29
Non-private practice^c^	0.24	1.27	0.84, 1.92	0.25
Central Canada region^d^	−0.09	0.91	0.75, 1.10	0.32
Eastern Canada region^d^	0.24	1.27	0.97, 1.68	0.087
Heard of ECC^e^	0.73	2.08	1.69, 2.56	<0.0001
Aware of CDA's position on ECC^e^	0.25	1.28	1.10, 1.50	0.002
Aware of their PDA's position on ECC^e^	0.25	1.29	1.10, 1.50	0.001

^a^ General dentist.

^b^ Male.

^c^ Solo private practice.

^d^ Western Canada region.

^e^ No.

A second logistic regression model was developed for dentists’ comfort in providing treatment for children with ECC (Table 7). Results showed a 2% reduction in the odds for being comfortable providing treatment with each additional year in practice (OR = 0.98, 95% CI: 0.98, 0.98; *p* < 0.0001). Pediatric dentists were 6.92 times more likely to report comfort in providing treatment than general dentists (OR = 6.92, 95% CI: 2.57, 18.61; *p* = 0.0001). Female dentists were 13% more likely to report comfort in providing treatment than male dentists (OR = 1.13, 95% CI: 1.03, 1.24; *p* = 0.007). Dentists practicing in clinics located in non-CMAs were 17% more likely to report comfort in providing treatment than those practicing in clinics located in CMAs (OR = 1.17, 95% CI: 1.07, 1.28; *p* = 0.0003). Dentists who reported having heard of ECC were 43% more likely to report comfort in providing treatment (OR = 1.43, 95% CI: 1.16, 1.76; *p* = 0.0007). Dentists who were aware of the CDA's position and their PDA's position on ECC were 26% (OR = 1.26, 95% CI: 1.13, 1.41; *p* < 0.0001) and 13% (OR = 1.13, 95% CI: 1.01, 1.25; *p* = 0.032) more likely to report comfort in providing treatment, respectively.

**Table 7:** Multivariate logistic regression model with participant characteristics and awareness of ECC for their comfort in providing treatment for children with ECC.

Variable	Regression coefficient	Odds ratio	95% CI for odds ratios	*p*-value
Intercept	1.95	–	–	–
Years in practice	−0.02	0.98	0.98, 0.99	<0.0001
Pediatric dentist^a^	1.93	6.92	2.57, 18.61	0.0001
Female^b^	0.13	1.13	1.03, 1.24	0.007
Group private practice^c^	−0.04	0.96	0.82, 1.13	0.66
Non-private practice^c^	0.13	1.14	0.87, 1.48	0.34
Non-CMA^d^	0.16	1.17	1.07, 1.28	0.0003
Heard of ECC^e^	0.36	1.43	1.16, 1.76	0.0007
Aware of CDA's position on ECC^e^	0.23	1.26	1.13, 1.41	<0.0001
Aware of their PDA's position on ECC^e^	0.12	1.13	1.01, 1.25	0.032

^a^ General dentist.

^b^ Male.

^c^ Solo private practice.

^d^ Census metropolitan area.

^e^ No.

## Discussion

The aim of this study was to analyze a subset of questions from a survey sent to Canadian dentists in 2013, relating to their awareness of ECC and views on prevention strategies for young patients and their caregivers.

### Dentist’s characteristics

Overall, 85.2% of survey respondents said they were comfortable with providing prevention services to children with ECC. However, fewer reported comfort in providing treatment to children with ECC (60.9%). It was found that practitioners who had been practicing for fewer years were more likely to be comfortable providing both prevention (*p* < 0.001) and treatment (*p* < 0.001) for patients with ECC.

Predictors for dentists being comfortable providing prevention and treatment for children with ECC included being in practice for fewer years, being a pediatric dentist, being a female dentist, having heard of ECC, being aware of the CDA's position on ECC, and being aware of the relevant PDA's position on ECC. Interestingly, practicing in a clinic located in a non-CMA was also a predictor for being comfortable providing treatment for children with ECC. It is understandable that more recent graduates are likely to have been taught about ECC within their dental school curricula, which would help to explain why they may be more comfortable providing prevention and treatment to children with ECC. Other studies have found that female dentists are more likely to see young children (19) and more likely to recommend having the first dental visit at a younger age (3, 18, 20) when compared to their male counterparts. This is likely related to female dentists typically showing greater empathy toward their patients than male dentists, as shown through their empathy and compassionate care scores (21). The CDA’ position on ECC emphasizes the importance of early childhood dental visits, risk assessment, use of topical fluoride, and anticipatory guidance and education for parents (22). Therefore, it is not surprising that those who were more aware of this position were more likely to be comfortable providing prevention and treatment for patients with ECC. The finding that dentists practicing in non-CMA based clinics were more likely to be comfortable treating children with ECC could be explained by these dentists understanding that there may be more limited access to pediatric dentists or possibly that their schedules and client pools allow them to see younger children.

### Practitioners’ knowledge and awareness of ECC

Due to the high prevalence of ECC, it is important that dentists, both pediatric and general, have expertise in providing oral health care for young children. Seeing patients at a young age helps to establish a dental home, setting a strong foundation for good oral health care throughout childhood and into adolescence (23). Results of this survey showed that while most Canadian dentists are familiar with ECC, there is still a significant proportion who are not as familiar as they could be. With more knowledge and experience of early childhood oral health care, dentists may be more likely to see young patients, specifically children of existing adult patients. However, another potential factor is a dentist's comfort and interest in treating young patients. If they are more interested, they may be more likely to be patient with uncooperative children and work with their parents to complete necessary treatments. Additionally, dentists that are more interested in treating young patients may be more likely to update themselves with current information/continuing education.

A recent study investigating what Canadian dental students are taught about early childhood oral healthcare showed that dental schools do have time in their curricula dedicated to teaching about the care of these age groups (24). However, oral health care of infants and toddlers is not listed as a core competency in over half of the dental schools across Canada. Additionally, less than a third of schools offered hands-on experience in performing assessments (24). This lack of hands-on experience is a potential causative factor of many dentists not feeling comfortable in providing preventive care and treatment to children with ECC. A potential solution to this issue is an interprofessional collaboration with primary care settings to expose undergraduate dental students to this population.

### Importance of early dental visits

There has been evidence that shows the benefits behind the recommendation of the first visit no later than by one year of age (25). Studies have found that the main factor in dental fear and/or anxiety (DFA) is past negative dental experiences (26). Therefore, early preventive dental visits where it is unlikely that any treatment will be required is a step in the right direction for preventing DFA (27). Also, the age at first visit has been shown to be associated with restorative treatment needed before the age of 6 years, with those children who are seen later more likely to need treatment (28). A recent study which investigated dentists’ views on a child's first dental visit found that the majority of Canadian dentists do not recommend the first visit to occur before one year of age (18). This could be because some dentists may not be comfortable providing oral health care services to children at a young age or may not feel that a first visit by 12 months is necessary (18). Little demand from parents for early visits may also contribute to this finding (18).

If a child's parent is a patient prior to the child's birth, prevention strategies may also be implemented much earlier. Dentists have an opportunity to talk to expecting parents about information surrounding early childhood oral health to start an infant's oral health on the right track. The perinatal period is an ideal time for oral health intervention, therefore maintaining good oral health and improving oral health knowledge during pregnancy is an effective first step in preventing ECC (8). Additionally, a recent systematic review reported a reduced incidence of ECC in children whose mothers received prenatal oral health care (9). This survey makes it apparent that only around two-thirds of Canadian dentists talk to expecting parents about infant nutrition, maternal nutrition, and the transmissibility of cariogenic bacteria. While it is encouraging that the majority cover these important topics, this proportion could still be improved. Our lab has previously reported that prenatal vitamin D levels are associated with ECC in infants (29, 30). Even though less than half of Canadian dental schools have time dedicated to teaching prenatal oral health care, all of them teach about the transmission of bacteria from caregiver to child (24).

### Need for education resources and treatment and prevention provided

It is encouraging to see that many respondents said they would like additional oral health resources designed to inform families on infant and toddler oral health and how to prevent ECC. This shows that dentists see the value in promoting infant and toddler oral health. It is also encouraging to see that almost two-thirds of Canadian dentists reported using fluoride varnish to manage ECC, especially when compared to a survey completed by Manitoban dentists in 2008, where only around 40% of dentists reported using fluoride varnish (3). It should be noted that another fluoride containing product, silver diamine fluoride (SDF), has demonstrated efficacy in the non-surgical management and prevention of ECC (31). However, it's use was not included in the survey since it had not yet been approved for clinical use. It was subsequently approved by Health Canada in 2017. The protocol for nonrestorative management of carious lesions in primary teeth has developed significantly since the time at which this survey was administered. For management of non-cavitated carious lesions, the use of sealants, 5% sodium fluoride (NaF) varnish, and 1.23% acidulated phosphate fluoride (APF) gel is recommended depending on the location of caries (e.g., occlusal). For nonrestorative management of cavitated carious lesions, biannual application of 38% SDF is currently recommended (32).

This study appears to be the first to investigate dentists’ views on ECC management and prevention on a Canada-wide level. Findings from this study coincide with those of similar studies. In a study of Malaysian general dentists, it was found that more recent graduates were more likely to recommend early dental visits and were more open to tolerating crying and uncooperative behaviour (33). Other studies have investigated dentists’ views on the recommended age for the first dental visit. In general, it appeared that while many were aware of the recommended age, not all dentists applied it in their practice. One study found that the majority of non-pediatric dentists in Ireland treated one infant per month, at most. This study also found that the majority of both pediatric and non-pediatric dentists did not receive training regarding infant oral health visits (34). Another study found that many mothers claimed that dentists would not accept their young children as patients until they were at least 3 years of age (35). Insight into dentists’ views on ECC management and prevention is a potential first step in resolving access to care barriers for this specific population.

This study was not without limitations. As mentioned, this survey was conducted 10 years ago. Since SDF had not been approved for clinical use until 2017 in Canada, this survey did not gather any information regarding dentists’ use of SDF for caries management. Additionally, the American Dental Association has since released evidence-based clinical guidelines to manage caries beyond the conventional restorative approach, which include treatment with 38% SDF (32). Therefore, any future surveys regarding prevention and management of ECC should investigate dentists’ use of SDF. Furthermore, as with many surveys, results were subject to response and recall bias. That is, it is likely that many respondents had a greater interest in preventive dentistry than those who did not respond, and survey responses were subject to respondents’ memories of their experiences, which may not always be accurate. While 3,232 dentists responded, some might consider this 21.9% of invited providers to be a modest response rate and may not fully represent the views of the entire dentist workforce in Canada.

## Conclusions

This study analyzed survey responses regarding early childhood oral health care from Canadian general and pediatric dentists. Results showed that while most Canadian dentists have sufficient knowledge of ECC, not all are comfortable providing oral health care services to children at a young age. However, it is encouraging that the majority of dentists talk to expecting parents about early childhood oral health care and that almost all dentists wanted more resources designed to inform parents about infant and toddler oral health care and ECC prevention.

## Data Availability

Publicly available datasets were analyzed in this study. This data can be found here: The dataset used and analyzed during this study was provided by the Canadian Dental Association, who own the data. Requests to access the datasets should be directed at: reception@cda-adc.
